# Pathogenesis of aerosolized Eastern Equine Encephalitis virus infection in guinea pigs

**DOI:** 10.1186/1743-422X-6-170

**Published:** 2009-10-23

**Authors:** Chad J Roy, Douglas S Reed, Catherine L Wilhelmsen, Justin Hartings, Sarah Norris, Keith E Steele

**Affiliations:** 1 Division of Microbiology, Tulane National Primate Research Center, Covington, Louisiana, USA; 2 Center for Vaccine Research, University of Pittsburgh, Pittsburgh, Pennsylvania, USA; 3 Office of the Commander, United States Army Medical Research Institute of Infectious Diseases, Fort Detrick, Maryland, USA; 4 Biaera Technologies LLC, Frederick, Maryland, USA; 5 Division of Pathology, United States Army Medical Research Institute of Infectious Diseases, Fort Detrick, Maryland, USA

## Abstract

Mice and guinea pigs were experimentally exposed to aerosols containing regionally-distinct strains (NJ1959 or ArgM) of eastern equine encephalitis virus (EEEV) at two exclusive particle size distributions. Mice were more susceptible to either strain of aerosolized EEEV than were guinea pigs; however, clinical signs indicating encephalitis were more readily observed in the guinea pigs. Lower lethality was observed in both species when EEEV was presented at the larger aerosol distribution (> 6 μm), although the differences in the median lethal dose (LD_50_) were not significant. Virus isolation and immunohistochemistry indicated that virus invaded the brains of guinea pigs within one day postexposure, regardless of viral strain or particle size distribution. Immunohistochemistry further demonstrated that neuroinvasion occurred through the olfactory system, followed by transneuronal spread to all regions of the brain. Olfactory bipolar neurons and neurons throughout the brain were the key viral targets. The main microscopic lesions in infected guinea pigs were neuronal necrosis, inflammation of the meninges and neuropil of the brain, and vasculitis in the brain. These results indicate that guinea pigs experimentally infected by aerosolized EEEV recapitulate several key features of fatal human infection and thus should serve as a suitable animal model for aerosol exposure to EEEV.

## Introduction

Eastern equine encephalitis (EEE) virus (EEEV) are a group of positive-strand RNA viruses in the genus *Alphavirus*, family Togaviridae, that cause significant morbidity and death in infected animals and humans [1-3]. The related alphaviruses Venezuelan equine encephalitis virus (VEEV) and western equine encephalitis virus (WEEV) also cause encephalitis and significant morbidity in humans and equines. Although naturally transmitted by mosquitoes, laboratory infections with these viruses [4] and experimental studies in animals have demonstrated that all three alphaviruses are infectious by the aerosol route and are considered a potential biowarfare threat.

Natural outbreaks of EEE have been reported primarily in North America; the South American varieties of EEEV appear to be less virulent in humans and animal models [5,6]. At a nucleotide level, South American strains in general are 25-38% different from North American strains [5]. In humans, the clinical disease and sequelae associated with EEEV infection are the most severe of any alphavirus, with an estimated mortality rate in humans of 30% for the North American strains [7]. The pathogenic effects of EEE in humans, horses, cows, and pigs have been clinically described in case reports from sporadic outbreaks in North and South America [[3,8], Jordan, 1965 #182, [9]]. In humans, EEEV infection results in a rapid onset of clinical signs and symptoms after exposure characterized by lethargy, hyperthermia, emesis, severe headache, and generalized malaise. Children, in particular, are susceptible. Pathologically, EEEV targets neurons in the brain, leading to widespread meningoencephalitis with neuronal necrosis, the formation of perivascular cuffs, and infiltration of the meninges and neuropil by neutrophils and mononuclear cells [7,10,11]. Vasculitis is also considered a key pathological feature of EEEV infection in humans. The disease rapidly progresses to severe hyperthermia, and coma, with death 5-15 days postinfection. Even among survivors, long-term neurological complications are common [11].

A variety of animal species have been investigated as possible disease models of human EEE. Limited pathogenicity studies using EEEV have been performed in pigs [12] and calves [13] using intraperitoneal, intranasal, or intracranial routes of exposure. Approximately 48-72 hr after exposure of those animal species, mild to moderate neurological signs appeared, manifesting as lethargy, somnolence, altered gait, incoordination, circling, recumbency, and paddling movements. Subcutaneous inoculation of EEEV is rarely fatal to adult mice; however, lethal infection can be induced in mice 3-5 weeks old [14,15]. Fibroblasts in the vicinity of the inoculation sites, osteoblasts in actively growing bone, and skeletal muscle myocytes are considered important cell types that support early viral amplification in young mice inoculated subcutaneously, but EEEV in mice has also has been shown to infect macrophages, dendritic cells, synovial cells, fibroblasts in several locations, keratinocytes, sebaceous glands, cardiac myofibers, retinal ganglion cells, and odontoblastic and ameloblastic epithelia of teeth [15]. Viremia develops rapidly, peaking at around 12 hr after subcutaneous inoculation and preceding neuroinvasion. Surprisingly, EEEV does not appear to enter into the brain through the olfactory bulb, as has been shown to be the case for VEEV and Sindbis virus infection [16-19]. Golden hamsters have been proposed as an additional disease model for natural EEEV infection, as they reportedly develop the vasculitis that is observed in human but not murine infections [20]. In cynomolgus macaques, aerosol exposure to EEEV results in fever and lethal encephalitis [21]. Virus isolation and immunohistochemistry indicated high viral titers in the brain and spinal cord. Vasculitis, which is reported in humans, was not seen in the macaques (Jo Lynne Raymond, unreported data).

Vaccines and therapeutics that protect against EEEV infection are needed to protect against the biowarfare threat posed by aerosolized EEEV. Demonstrating efficacy against aerosol exposure to EEEV would not be possible in a human clinical trial as inhalation is not a natural route of EEEV transmission. Licensure is only possible through the FDA's 'Animal Rule', which allows for the demonstration of efficacy in multiple well-characterized, relevant animal models [22]. Because experimental studies have demonstrated antigenic differences between North American and South American strains of EEEV, it will be important to demonstrate protection against strains from both geographic origins in suitable animal models [6]. A survey of the available disease models for EEEV revealed that few available studies address animal species susceptibility or regional viral strain virulence in experimental aerosol infection. Further, there are no reports of differential pathogenicity of aerosolized EEEV when presented at distinctly different particle size distributions in any laboratory animal species.

The objective of this study was to initially assess the susceptibility of mice and guinea pigs to aerosolized EEEV and to describe the resulting pathology associated with experimental infection. Initially, to assess susceptibility and derive lethality estimates, groups (n = 8) of mice and guinea pigs were exposed by aerosol using either a North (NJ1959) or South American (ArgM) strains of EEEV presented at two distinctly different particle size distributions. We performed five target doses (x5) per viral strain (x2) per species (x2) per aerosol size distribution (x2) for a total of 40 individual experiments. Thereafter, groups of guinea pigs were exposed to either strain of EEEV (x2) at either aerosol size distribution (x2) to further distinguish pathology that may have resulted from multimodal aerosol exposure.

## Materials and Methods

### Animals

Female BALB/c mice and Hartley guinea pigs of both sexes were obtained from Charles River Laboratories (National Cancer Institute, Fort Detrick, MD). The mice were approximately 19-22 g and 8-9 weeks old; the guinea pigs were 8-10 weeks old and approximately 250 g at the time of exposure. Animals were provided with rodent chow and water *ad libitum *and maintained on a 12 hr light/dark cycle.

### Reagents & Virus

North American (New Jersey 1959; NJ1959) and South American (Argentina M; ArgM) strain of the EEEV were used in the experimentation. Viruses were propagated in cell culture as previously described [23]. Viral titer was assayed by a Vero cell plaque assay (described below), and was diluted to make the appropriate concentrations for the aerosol experiments. The viral stock was diluted with Eagles minimal essential medium (EMEM) (pH = 7.4) before aerosolization and 10% sterile glycerin was added to generate larger particles. Glycerin also increased the viscosity of the suspension, which facilitated the formation of larger particle aerosols in those series of exposures [24].

### Exposure System and Aerosol Generation

Mice were exposed to EEEV aerosols in whole-body exposure chambers housed within Class III biological safety cabinets maintained under negative pressure (-1 WC"), as previously described [24]. The animals were exposed inside a whole-body chamber which could contain up to four smaller stainless steel mesh restraint cages holding approximately 10 mice/cage or two guinea pigs/cage. The animal exposures were acute and lasted only 10 min. A Collison nebulizer (BGI Inc., Waltham, MA) was used to generate the smaller (1 μm) particles. A spinning-top aerosol generator (STAG) was used to generate the larger particles (BGI USA, Waltham, MA). Total flow through the inhalation system was 19.0 ± 0.5 liters per min (LPM) during the exposures created by the Collison nebulizer; total flow measured 22 ± 0.5 LPM when the STAG was in use. The metrics for using both of these generation devices in association with the inhalation system was previously described [24]. The test atmosphere in both systems was sampled during the exposure for size characterization and aerosol concentration as was previously described. Results of the particle sizing with the Aerodynamic Particle Sizer 3321 (TSI, Inc, St. Paul, MN) indicated a mass median aerodynamic diameter (MMAD) of 1.0 μm and geometric standard deviation (σ_g_) of 1.3 for the aerosols generated by the Collison nebulizer. The size distribution for the particles generated by the STAG was summed as being composed of distinctly 'larger' particles (> 6 μm) than the Collison-generated aerosols. Exposure concentration, expressed in plaque-forming units (PFU)/l, was determined by isokinetic sampling of the chamber with an all-glass impinger (AGI; Ace Glass, Vineland, NJ). EMEM medium with antifoam A 0.001% w/v (Sigma, St. Louis, MO) was used to collect medium in the impinger. 'Presented' dose was estimated by calculating the respiratory minute volume (Vm) using Guyton's formula (Guyton, 1947), expressed as Vm = 2.10 × W_b _^0.75 ^where W_b _= body weight (g), based upon the average of group weights the day of exposure. The presented dose was then calculated by multiplying the estimated total volume (Vt) of experimental atmosphere inhaled by each animal (V_t _= V_m _× length of exposure) by the empirically determined exposure concentration (C_e_) ('presented dose' = C_e _× V_t_).

### Plaque Assay

Viral titers in the aerosol generator starting suspensions, AGI samples, tissues, and blood were determined by plaque assay as previously described. Tissues obtained at necropsy were ground with a sterile mortar and pestle and a 10% homogenate v/w solution was prepared before viral titration. Briefly, for plaque assay, samples of homogenized tissue or blood were diluted with a solution of EMEM with 5% fetal bovine serum (FBS) and gentamycin (without HEPES) added to 6-well plates containing a confluent monolayer of Vero-E6 cells (green monkey kidney cells) which were incubated at 37°C for 1 hr. Then, a 0.5% agarose overlay in 2× basal medium EMEM with Earle's salts (EBME) solution (with HEPES salts and 5% FBS and gentamycin) was added, and plates were incubated at 37°C at 5% CO2 for 48 hr. Thereafter, a second overlay of saline A (SA) with 5% neutral red and 5% FBS was added, and the plates were again incubated at 37°C for 4 hr. Defined plaques (neutral red exclusion areas) were then counted.

### Necropsy & Pathology

Guinea pigs were euthanized at the prescribed time points in the second experiment by administering an overdose of Euthasol^® ^(pentobarbital sodium and phenytoin sodium; Delmarvia Laboratories, Midlothain, VA). A complete necropsy was conducted on each guinea pig. Heart blood samples were collected aseptically into pediatric EDTA-anticogulant evacuated tubes. Samples of cerebrum, lung, and liver were then obtained aseptically, placed in sterile centrifuge tubes, and stored at -70°C until processing for virus isolation. Additionally, a full set of tissue samples were collected for histopathology. These tissues were fixed in 10% neutral buffered formalin and stored for a minimum of 21 days within biosafety (BSL)-3 containment to ensure all virus had been deactivated before histopathology processing. Bones were decalcified in formic acid, and all tissues were trimmed, routinely processed, and embedded in paraffin. Tissue blocks were sectioned at 5-6 μm on a rotary microtome and mounted on glass slides. All tissue sections were stained by hematoxylin and eosin. In addition, duplicate sections were prepared for immunohistochemistry from the lungs, submandibular lymph nodes, haired skin, salivary glands, brain, and multiple tissues of the skull. Immunohistochemistry was performed as described previously [25], with modifications. Briefly, sections were deparaffinized and rehydrated. Antigen retrieval was performed by immersing slides in citrate buffer, pH 6.0, for 30 min at 97°C and endogenous peroxidase was blocked. Sections were incubated with the primary antibody, a polyclonal rabbit antiserum directed against EEEV, WEEV, VEEV and Sindbis virus (supplied by Ms. Cindy Rossi, USAMRIID) diluted 1:10,000, for 30 min at room temperature. The remainder of the procedure utilized a peroxidase-labeled secondary antibody and chromogen from a commercial kit (Envision System, DAKO Inc., Carpinteria, CA) according to the manufacturer's instructions. Sections were then rinsed, counterstained with hematoxylin, dehydrated, and covered with a coverslip. Non-immune (normal) rabbit serum was used as a negative control for the primary antibody.

### Statistical Analysis

Dose-response curves were constructed and LD_50 _values were determined by probit analysis using PROC PROBIT in SAS Version 9.1.3 (SAS Institute Inc., Cary, NC, 2007). Times-to-death were compared by Wilcoxon rank-sum tests. Descriptive statistics (mean ± standard deviation) were used to display the results of the tissue and blood viral titers.

## Results

### Lethality of aerosolized EEEV in mice and guinea pigs

**Figure 1:**
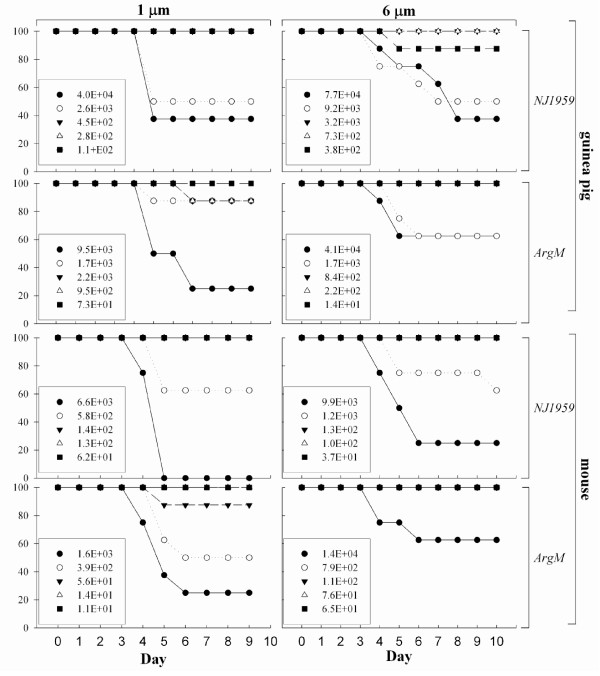
**Survival of guinea pigs and mice after aerosol exposure to EEEV**. Guinea pigs (top graphs) and mice (bottom graphs) were exposed to different doses of NJ1959 (first and third row graphs) or ArgM (second and fourth row graphs) at 1 μm (left column) or > 6 μm particle sizes (right column). Graphs show percent survival (*y *axis) for each group for each day postchallenge (*x *axis).

To examine the lethality of EEEV strains when aerosolized, Hartley guinea pigs and Balb/c mice were exposed to either large (> 6 μm) or small (~1 μm) particle aerosol distributions containing the ArgM or NJ1959 strains of EEEV (figure 1). Mice and guinea pigs succumbed to the infection between days 4-8 postexposure (PE) to either strain of EEEV; there were no significant differences in time to death between the viral strain, particle size, or dose of EEEV in either mice or guinea pigs. A generalized flaccid paralysis was observed in the mice that received the higher doses (> 1,000 PFU) of the virus, although specific signs were difficult to distinguish in this species. In guinea pigs, the initial clinical signs of EEEV infection, including limited motility and dorsal tremor first appeared (18-24 hr PE) in the guinea pigs exposed to the larger particle aerosols. Similar signs of infection were not observed in the guinea pigs exposed to the smaller distribution (1 μm) until 65-72 hr PE. Once initial signs of viral infection began in guinea pigs, the disease progression was similar for all exposures, and included head tilt, severely altered gait, and loss of equilibrium. Clinical signs rapidly progressed to circling, lateral recumbency, and paddling motions of the forepaw limbs, which culminated with coma and death.

Analysis of the survival data indicated that the virulence of aerosolized EEEV was greater in mice than in guinea pigs (Table 1). Probit analysis of the survival data indicated that for mice exposed to 1 μm particle size aerosols containing EEEV there was no significant difference in the lethality of NJ1959 and ArgM (p = 0.1032). At a particle size > 6 μm, the calculated LD_50 _for NJ1959 was 2.9 × 10^3^, almost five times the LD_50 _for NJ1959 at 1 μm (6.2 × 10^2^) although this difference was also not significant (p = 0.1057). An LD_50 _could not be determined for the ArgM strain at a particle size > 6 μm because mortality was not ≥ 50% in any of the dose groups.

**Table 1:** Particle size and viral strain specific LD_50 _determination, by speciess

**Species**	**EEE strain**	**Particle (μm)**	**LD_50 _(PFU)**	**95% fiducial limits**		**MTD^a ^(days)**
					
				**lower**	**upper**	
guinea pig *Hartley*	ArgM	1	5.2E+03	2.9E+03	2.4E+04	6.0
	
		> 3^b^	n/c^c^	-^d^	-	5.0
	
	NJ1959	1	1.1E+04	3.5E+03	8.9E+04	5.0
	
		> 3	3.0E+04	9.1E+03	7.6E+05	5.8

mouse *BALB/c*	ArgM	1	4.6E+02	1.9E+02	1.7E+03	6.0
	
		> 3	n/c^c^	-	-	4.7
	
	NJ1959	1	6.2E+02	-	-	5.8
	
		> 3	2.9E+03	1.1E+03	1.1E+04	5.8

^a ^Mean time to death

^b ^Considered 'larger' particle distribution due to bimodal size distribution

^c ^LD_50 _could not be estimated because no dose group had more than 50% mortality

^d ^The confidence limits were not determined due to lack of mortality in selected groups

For guinea pigs, the LD_50 _of 1 μm aerosol particles containing NJ1959 was over 17 times higher than what was calculated for mice (1.1 × 10^4 ^compared to 6.2 × 10^2^, respectively) (p = 0.0098). The LD_50 _for the > 3 μm particles containing NJ1959 was not significantly different from the LD_50 _for 1 μm particles of NJ1959 (p = 0.3500) which is similar to what was observed in the mouse exposures. The LD_50 _for ArgM at 1 μm was half that of NJ1959 (5.2 × 10^3 ^compared to 1.1 × 10^4^, respectively) but an LD_50 _could not be calculated for > 6 μm ArgM particles as ≥ 50% mortality was not observed in any of the dose groups.

### Pathogenesis of aerosolized EEEV in guinea pigs

**Figure 2:**
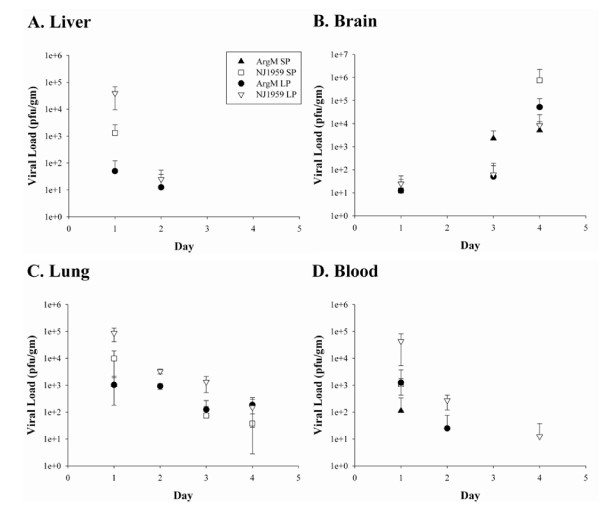
**Viral load in guinea pig tissues after aerosol exposure to EEEV**. Guinea pigs were exposed to aerosolized EEEV strains NJ1959 (open symbols) or ArgM (filled symbols) at a dose of 1 × 10^6 ^PFU; each day four guinea pigs from each group were necropsied and samples of liver (*A*), brain (*B*), lung (*C*) or blood (*D*) were taken for virology. Graphs showed averaged viral load in PFU/g ± the standard deviation for each day postexposure.

To better define the pathogenesis of aerosolized EEEV in the guinea pigspecies, we infected groups at a dose of 1.0 × 10^6 ^PFU of either NJ1959 or ArgM at 1 μm or > 6 μm particle distributions. Thereafter, the guinea pigs were euthanized on days one, two, three or four postexposure and necropsied to collect tissues for histological examination and for virus isolation. Four guinea pigs were sampled at each time point for each viral strain/particle size combination. Regardless of viral strain or particle size, virus was isolated from all four tissues examined (liver, lung, brain, and blood) one day after challenge (figure 2). Virus was only detectable on days one and two postexposure in the liver and blood except in guinea pigs exposed to > 6 μm particles of NJ1959 where virus was found in the blood on day four. For 1 μm exposures, the levels of NJ1959 were higher than ArgM on day one in the liver, lungs, and blood although these differences were not statistically significant. Viral titers in the lungs remained fairly constant throughout the four days postexposure, declining slightly on days three and four. In contrast, the level of virus in the brain increased rapidly throughout the four days examined for both viral strains and particle distributions, from 10^1 ^PFU/mg on day 1 to 10^4^-10^6 ^PFU/g on day four. The level of virus in the brain on day four was highest in the guinea pigs exposed to the 1 μm aerosols containing NJ1959.

**Figure 3:**
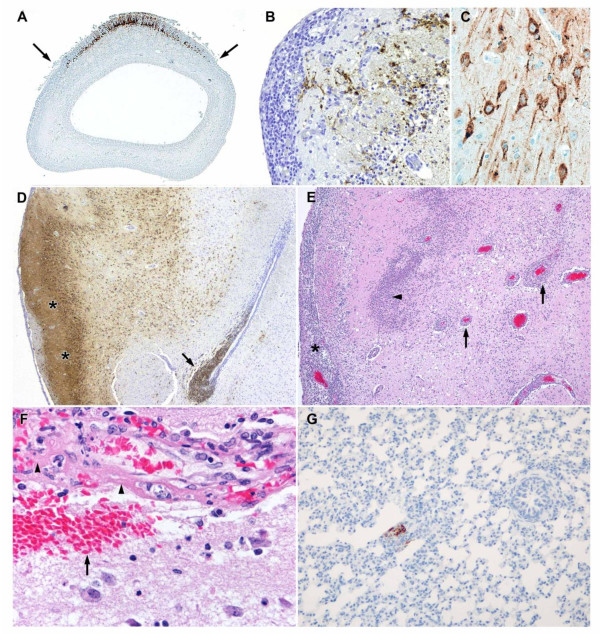
**Pathogenesis of EEEV in guinea pigs after aerosol exposure**. (*A*) EEEV antigen was present in a segment of olfactory neuroepithelium, but not in the adjacent respiratory epithelium, of a guinea pig 48 hr after exposure. The junctions of olfactory mucosa and respiratory mucosa are demonstrated by the arrows. (*B*) EEEV antigen is present in the olfactory bulb of a guinea pig 72 hr after exposure. Note the disruption of the neuropil and the infiltration of the neuropil and meninx by inflammatory cells. (*C*) EEEV antigen was present in the cell bodies and neuritic processes of several neurons in the brain of a guinea pig 72 hr after exposure. (*D*) In the brain of the same guinea pig as in Figure 3C, there is abundant viral antigen throughout the pyriform cortex (*'s) and the deeper basal nucleus, as well as in the periventricular cells (arrow). (*E*) Abundant inflammatory cells thicken the meninx overlying the pyriform cortex (*), form cuffs around blood vessels in the basal nucleus (arrows) and also infiltrate the neuropil (arrowhead) of a guinea pig 96 hr after exposure. (*F*) In the brain of the same guinea pig as in Figure 2E, the wall of an inflamed blood vessel is disrupted, infiltrated by inflammatory cells and contains eosinophilic (fibrinoid) material (arrowheads). Also, note the perivascular hemorrhage (arrow). (*G*) Minimal viral antigen is present in the lung of a guinea pig 48 hr after exposure.

Pathology data indicated that productive viral infection of tissues of infected guinea pigs was almost exclusively limited to the olfactory segment of the nasal tract and the brain. Infection of the olfactory segment of the nasal tract was evident in 11/16 (69%) guinea pigs at day one postexposure by positive immunohistochemistry of the olfactory mucosa, the olfactory nerves, and/or the lamina propria. Both viral strains and both particle sizes resulted in infection of the olfactory tract at this time. Affected guinea pigs had one or more foci of immunolabeled olfactory mucosa scattered throughout the nasal tract, often associated with positive olfactory nerves and lamina propria. The foci that contained EEEV antigen were without apparent morphological changes in infected cells of all cases at day one. At day two postexposure, the olfactory tracts of 15/16 (94%) guinea pigs exhibited positive immunohistochemical staining for viral antigen (Figure 3A). In individual cases, there was more extensive labeling of EEEV antigen in the olfactory tract than was evident in any of the cases euthanized at day one postexposure. However, some guinea pigs had only minimal antigen at day two and even those with the most abundant antigen labeling demonstrated infection of only a minor percentage of the olfactory neuroepithelium. Many of the infected cells were identified as olfactory neurons by the presence of viral antigen within both the cell body and the apical processes. Basal cells also contained antigen in some cases, as did apparent macrophages and fibroblasts in the lamina propria. The possibility that sustentacular cells were also infected could not be ruled out, but it was not possible to identify these cells morphologically in immunostained slides. Another feature apparent in some animals was positive immunostaining of segments of olfactory mucosa that ended abruptly at the junction with respiratory mucosa. The respiratory epithelium was uniformly negative in all guinea pigs. This finding implied a tropism of aerosolized EEEV for olfactory cells but not respiratory cells. At day two postexposure, some immunolabeled foci of olfactory mucosa had a few degenerate or apoptotic cells and minimal infiltrates of heterophils; however, most positive foci remained morphologically unaffected at the light microscopy level. The majority of guinea pigs euthanized at days threee and four postexposure had small amounts of EEEV antigen in the olfactory tract. At these time points, the morphological changes in affected olfactory tract were more prominent than on day one or two postexposure and included focal to segmental degeneration and cell death in the olfactory mucosa and infiltration of affected foci by heterophils. Affected cells in the olfactory mucosa were characterized by peripheral nuclear condensation, nuclear fragmentation, and the presence of apoptotic bodies in the olfactory mucosa, indicating that a significant proportion of the dead cells had undergone apoptosis.

Infection of the brains of guinea pigs was first evident by the presence of limited EEEV antigen within the lamina fibrorum and lamina glomerulosa of the olfactory bulbs of 5/16 (31%) guinea pigs at day one postexposure. Histologically, the olfactory bulbs did not exhibit abnormal changes on day one. At day two postexposure, abundant viral antigen was present throughout all layers of the olfactory bulbs of all guinea pigs and the meninges of the olfactory bulbs were minimally to mildly thickened by infiltrates of macrophages, lymphocytes, and some heterophils at this time point and at later time points (Figure 3B). There was minimal hemorrhage in the olfactory bulbs of a few guinea pigs at day two postexposure. Virus antigen was also present in the brain proper of 15/16 (93%) guinea pigs at day two postexposure. Antigen, primarily in neurons, was limited to the olfactory structures in the rostral brain at this time, including the olfactory tubercles, olfactory nuclei, and pyriform cortices. The meninges overlying the olfactory portion of the brain in several animals contained minimal infiltrates of macrophages, lymphocytes, and some heterophils. At day three postexposure, abundant viral antigen was present in the olfactory nuclei and pyramidal layer of the olfactory tubercles and in the pyriform cortices of all animals, again predominantly within neurons (Figure 3C). In addition, there was often extensive viral antigen in the periventricular cells of the lateral ventricles (Figure 3D). Less frequently, neurons in the neocortex, thalamus, hippocampus, and basal nuclei were positive by immunohistochemistry. At day 3, there was infiltration of the neuropil in the pyramidal layer of the olfactory tubercles and pyriform cortex by numerous heterophils, many of which were degranulated and/or degenerate. Neurons in affected areas appeared shrunken and a few had hypereosinophilic cytoplasm. Blood vessels in these areas and in the basal nuclei were often lined by hypertrophied endothelium and the Virchow-Robbins space contained macrophages and heterophils that in some cases formed cuffs of up to three cell layers. The walls of a few blood vessels were disrupted or fragmented and were infiltrated by viable and degenerate inflammatory cells mixed with erythrocytes, indicating early vasculitis. The meninges of the olfactory tubercles and pyriform cortex at this time were edematous and contained infiltrates of macrophages, heterophils and lymphocytes. Meningeal inflammation extended from the olfactory portion of the brain locally into the neocortex in some cases. At day four postexposure, viral antigen was again most extensive in the olfactory structures of the brain, but was increased in other locations as well. Antigen was seen in large motor neurons of the caudal brainstem of 3/16 (19%) animals and in Purkinje neurons of the cerebellum of 2/16 (12%) animals. At day four, many pyramidal neurons in the olfactory tubercles and pyriform cortex were necrotic and these areas had extensive infiltration by heterophils with fewer mononuclear cells, admixed with cellular debris (Figure 3E). There was vasculitis in these areas, and also in the basal nuclei, septum, and neocortex that was more severe than at day three. Rarely, there was fibrinoid necrosis of vessels (Figure 3F) at day 4 PE. The neuropil also contained scattered microhemorrhages at day four and there was prominent inflammation surrounding the lateral ventricles in animals in which periventricular antigen was present. In addition to neurons, a few cells that appeared to be glial cells, degenerate neurons, or possibly inflammatory cells contained viral antigen. Ependymal lining cells rarely contained antigen, and antigen was present in cells in the lumens of some lateral ventricals that appeared to be macrophages.

Neurons were the primary target of EEEV in the brains of infected guinea pigs. In particular, EEEV showed tropism for medium to large neurons such as pyramidal neurons in the neocortex, Purkinje neurons, and motor neurons in the brainstem. In addition, some scattered smaller cells in the brain, possibly small neurons and/or glial cells, also contained viral antigen, as did some apparent inflammatory cells and the previously mentioned periventricular cells. Neither the strain of EEEV, nor the size of aerosol particles with which guinea pigs were infected appeared to have any effect on how rapidly virus appeared in the olfactory mucosa or the brain. However, at day two, subjectively there did appear to be slightly more antigen in both the olfactory mucosa and rostral brain of guinea pigs infected with large particle aerosols than with small particle aerosols.

Minimal amounts of viral antigen were present in the lungs of some guinea pigs infected with the NJ1959 strain of EEEV (Figure 3G), but viral antigen was not evident in any of the animals infected with the ArgM strain. In the guinea pigs infected with the large particle NJ1959 strain, only a single animal, euthanized at day one postexposure, had minimal viral antigen in the lumen of a small airway. In those animals infected with the small-particle NJ1959 strain, all four animals at day one postexposure had viral antigen in the lungs and two of the four animals at day two had positive lungs. The lungs of the remaining animals were negative which included the lungs of all 32 guinea pigs euthanized at days three and four. In the positive lungs, viral antigen was present either in the epithelium lining conducting airways measuring 50-150 μm, interpreted as small bronchi or bronchioles, or in alveoli. Some of these foci exhibited degeneration or apoptosis of the few positive cells and some affected cells were sloughed into the lumens of small airways. These were the only histological changes that we associated with EEEV infection of the lungs; however, the lungs of many of the guinea pigs contained scattered foci of macrophages, lymphocytes, and some heterophils consistent with inflammation due to inhaled particulate material, i.e., pneumoconiosis.

Additional immunohistochemistry findings included rare positive cells that appeared to be osteoblasts in the skulls of four guinea pigs, small foci of positive subgingival or periodontal connective tissue in three cases and a focus of positive odontoblastic epithelium in a single case. Otherwise, the structures of the head, including the mandibular lymph nodes, the nasal-associated lymphoid tissues, and the vomeronasal organs, did not exhibit positive immunostaining for EEEV antigen, nor was there histological evidence of viral infection of other tissues. In particular, there was no evidence of viral infection of the respiratory lining cells of the nasal tract, nor the tracheas, of any guinea pigs in the study. Evidence of EEEV infection of other tissues, including the heart, liver, spleen, kidneys, adrenal glands, stomach, small and large intestines, pancreas, urinary bladder, testes, ovaries, uterus, skin, salivary glands, thymus, bone marrow, and mediastinal and mesenteric lymph nodes, was uniformly lacking. In addition to pneumoconiosis, lesions considered to be common findings in guinea pigs were seen in some cases. Changes consistent with vitamin C deficiency were evident in the tibia and femur of most guinea pigs in the study. In several guinea pigs, rhabdomyomatosis was present in the heart and/or mild changes consistent with early segmental nephrosclerosis were present in the kidneys.

## Discussion

This study was performed to further assess the relative infectivity of EEEV as an aerosol, to assess the comparative virulence of representative North and South American strains of EEEV when aerosolized, and to initiate characterization of rodent models of aerosol disease to be used in future testing of medical countermeasures against EEEV. In the initial exposure experiments, lethality estimates were established in both animal species, although mortality in the groups did not consistently match the increase in dose received. This variation may account for the lack of fiducial limits in some of the probit analysis. In a previous pathogenesis study using juvenile albino mice inoculated intracranially or subcutaneously with EEEV strains E-1 and P-7, a sharp dose-response curve was observed, although the doses were simply reported as an inoculating volume of 20% brain suspension homogenate from experimentally infected suckling mice, which allows no basis for comparison [14]. Other subsequent investigative studies with intracranially inoculated pigs failed to establish a dose-response relationship with the virus [12]. The clinical signs of animals in the current study, manifesting as partial to complete paralysis in the mice and asymmetrical neurological signs in the guinea pigs, temporally matched the similar onset of signs observed in intracranially infected domestic pigs. Clinical signs in a single calf experimentally infected with EEEV-infected brain homogenate were similar to those observed in the rodent and pigs, including nystagmus and dorsal tetanic spasm, 3 days after onset of disease [13].

In this study, the guinea pigs proved to be the better model for EEEV infection than mice, in that they developed more uniform clinical signs with an easily identified onset and lethality in the lethal dose determination. Later stages in the clinical illness (coma, death) illustrated what is typically observed in encephalitic syndromes caused by neutrotropic viruses. Clinical signs were difficult to assess in mice until very late in the course of the disease. Hamsters are the alternative rodent model for studying EEEV infection. At this time, we cannot distinguish whether guinea pigs or hamsters are the superior model since both develop pathologies similar to what has been reported to humans, particularly the development of vasculitis [20]. However, it should be noted that guinea pigs and hamsters were infected by different routes of exposure (aerosol exposure and subcutaneous inoculation, respectively) which can alter the pathogenesis of the disease. Because guinea pigs have been evaluated for aerosol exposure, we believe them to be the superior model for evaluating the efficacy of candidate vaccines or therapeutics for the aerosol route of exposure.

It was expected that particle size distribution would alter the virulence and pathogenesis of aerosolized EEEV. The anatomical differences in the respiratory tract of a guinea pig when compared to a mouse would affect particle deposition and subsequent initiation of disease. Larger aerosol particles of EEEV with a MMAD of > 6 μm would be expected to deposit more in the upper respiratory tract, leading to infection of the olfactory neuroepithelium and the olfactory bulb and more rapid penetration into the brain of exposed animals than would be the case for aerosol exposure to EEEV in a smaller, more highly respirable particle. Exposure to a larger particle size would be expected to lower the dose required to cause disease and potentially accelerate the progression of the infection [24]. It was surprising then, that the LD_50 _for both strains of EEEV was higher for aerosol exposures at the larger particle size and could not be determined for the ArgM strain in mice. We pursued the serial sacrifice studies in the guinea pig model to examine the pathogenesis of aerosolized EEEV infection as a result of these initial findings in the lethality estimation experiements

Our observation of early infection of the olfactory neuroepithelium seems to represent a cardinal feature in the pathogenesis of aerosolized EEEV in guinea pigs. In particular, EEEV infection of the so-called bipolar olfactory neurons is key, because at one 'pole' these cells have ciliary processes that extend into the air passages, where they likely contact virus, and at the opposite 'pole' they extend axons that synapse directly with neurons in the olfactory bulbs. Viruses that target the olfactory neurons therefore have a direct portal to the brain. The additional finding of early infection of olfactory neurons in guinea pigs exposed to both small- and large-particle aerosols, however, fails to explain why the guinea pigs exposed to large particle aerosols had higher LD_50_. Given the observed infection of the olfactory neurons by EEEV, it was not unexpected that infection of the brain was first evident as viral antigen within the olfactory bulbs and other olfactory brain structures. This was followed by apparent transneuronal spread to other parts of the brain in generally a rostral to caudal fashion, reaching the caudal brainstem and cerebellum of a few guinea pigs by day four which was the last day studied. Viral infection of the brain resulted in direct damage to neurons as well as widespread meningoencephalitis. These important pathogenetic features, infection of olfactory neuroepithelium, invasion of the olfactory brain, transneuronal spread, direct neuronal cytopathicity, and inflammation of the brain, are essentially identical to the mouse model of aerosolized VEEV, to date the best characterized animal model of aerosolized alphavirus infection [2,17-19].

There were some differences between the mouse model of aerosolized VEEV and the characteristics of EEEV infection seen in our current studies with guinea pigs. Exposure to aerosolized VEEV causes massive infection of the olfactory neuroepithelium in mice, [18,19] whereas aerosolized EEEV infection was apparent only in a few usually small foci of the olfactory neuroepithelium of guinea pigs. This finding in guinea pigs is similar to previous studies in our laboratory with attenuated VEEV strains in which limited productive infection of the neuroepithelium by the attenuated viruses was nonetheless sufficient to result in viral entry into the brain [18]. This aspect is relevant to efforts to develop vaccines effective against aerosolized alphaviruses and punctuates that even small amounts of virus that might evade local immune mechanisms in the olfactory tract could be sufficient to infect the brain. Another difference noted in this study is that productive infection due to aerosolized EEEV was apparent only in the olfactory neuroepithelium and the brains of guinea pigs, whereas VEEV infection of mice appears to have somewhat broader tropism, consistently infecting Bowman's glands, the teeth, vomeronasal organs, the pancreas and, importantly, lymphoid tissues. Neither EEEV in this study, nor VEEV in previous studies, appeared to result in sustained infection of the respiratory epithelia in the lungs and upper respiratory tract of guinea pigs and mice, respectively. This particular feature is likely of little importance however, given the significant susceptibility of the olfactory neuroepithelium, and the consequences thereof, as previously discussed.

There were also similarities and differences between the guinea pigs in this study and other animal models of EEEV that utilized peripheral inoculation of this virus. Key among the similarities, cutaneous inoculation of young mice [14,15] and golden hamsters [20] with EEEV resulted in neuroinvasion, targeting of neurons, and widespread infection of the brain leading to death. However, in both the mice and hamsters infected with EEEV cutaneously, virus appeared in multiple regions of the brain simultaneously, indicating neuroinvasion by a vascular route. This is in contrast to the guinea pigs in our study, in which neuroinvasion appeared to occur via the olfactory tract. A reported feature of golden hamsters infected with EEEV which is possibly relevant to human infections with EEEV is that, unlike mice, they develop vasculitis [20]. We also observed vasculitis as a late-developing feature of meningoencephalitis in the guinea pigs inoculated with aerosolized EEEV. However, the significance of vasculitis relative to human cases of EEEV is uncertain. While vasculitis can certainly contribute to the overall brain damage that occurs in EEEV, it may be of limited consequence with regard to the ultimate cause of death, especially given that neurons are collectively an overwhelming target of viral infection and appear to suffer from direct viral damage.

Two other particular features of EEEV infection of the guinea pigs in this study merit discussion. First, we did not observe tropism of EEEV for lymphoid tissues and little, if any, viral antigen within macrophages in any tissues. Studies with mice and hamsters also reported limited lymphoid tropism by EEEV [15,20]. These findings are in contrast to findings in experimental animal models with VEEV, where that virus is strongly tropic for lymphoid tissues and causes severe lymphoid necrosis [2,18,19,26]. Infection of macrophages may be central to the lymphoid damage caused by VEEV [27]. Hamsters and guinea pigs, as well as rabbits, develop such severe necrosis of lymphoreticular tissues after inoculation with VEEV that they die from bacterial overgrowth and endotoxic shock before full manifestation of central nervous system infection [2,26]. This has significantly limited the utility of these three species as experimental models of VEEV. The lack of lymphoid tropism exhibited by EEEV is therefore presumably necessary for the manifestation of neurological infection in hamsters and guinea pigs when virus is inoculated peripherally. The second feature of EEEV infection of guinea pigs worth discussing regards the matter of infection of osteoblasts. It was recently reported that osteoblasts are an important early target of EEEV in young mice and it was postulated that this might contribute to the greater sensitivity of the young to infection with this virus [15]. We also observed a few cells with EEEV antigen in the skulls of guinea pigs that appeared to be osteoblasts; however, these cells were rare. This difference in osteoblast susceptibility between guinea pigs and mice infected with EEEV could be due to species differences, the ages of the animals used in the two studies or other factors. A variety of additional pathogenetic features in animal models of alphavirus infection have also been described, but additional comparisons to those studies are beyond the scope of this report.

An additional aim of this study was to determine the virulence and pathogenesis of a South American strain of EEEV. South American strains of EEEV are antigenically distinct from North American strains and vaccines against North American strains may not be protective [6]. South American EEEV strains are thought to be less virulent than North American strains; however, enzootic subtypes of VEEV that cause only mild disease by subcutaneous inoculation can cause significant morbidity and encephalitis in animals when aerosolized [28]. This variation in virulence as a result of exposure raised concern that South American strains of EEEV might also be more virulent by aerosol. The results reported here indicate that a South American strain of EEEV was as virulent by inhalation as a North American strain that has caused disease in humans. Therefore, vaccines or therapeutics developed to protect against aerosol exposure to EEEV should be evaluated against both North American and South American strains.

In summary, mice and guinea pigs are susceptible to lethal infection by North and South American strains of EEEV. The guinea pig model of aerosolized EEEV recapitulates several key features of fatal human infection by EEEV. It also shares several important pathogenic features with aerosolized VEEV infection in mice. Thus, guinea pigs should serve as a useful animal model for aerosol exposure to alphaviruses in general, and for EEEV in particular.

## Ethics

Research was conducted in compliance with the Animal Welfare Act and other federal statutes and regulations relating to animals and experiments involving animals and adheres to principles stated in the *Guide for the Care and Use of Laboratory Animals*, National Research Council, 1996. The facility where this research was conducted is fully accredited by the Association for Assessment and Accreditation of Laboratory Animal Care International.

## Competing interests

The authors declare that they have no competing interests.

## Authors' contributions

CJR and DSR performed the experiments, JMH assisted with the engineering performance of the experiments. SN participated in the design of the study and performed the statistical analysis. CLW and KES performed pathological analysis. CJR conceived of the study, and DSR participated in its design and coordination. All authors read and approved the final manuscript.
